# Metabolic profiling, antimicrobial, anticancer, and in vitro and in silico immunomodulatory investigation of *Aspergillus niger* OR730979 isolated from the Western Desert, Egypt

**DOI:** 10.1007/s10123-024-00503-z

**Published:** 2024-03-20

**Authors:** Amira M. Baz, Esmat Elwy, Wafaa A. Ahmed, Heba El-Sayed

**Affiliations:** 1 Botany and Microbiology Department, Faculty of Science, Cairo University, Giza, Egypt; 2 Biochemistry and Molecular Biology Unit, Department of Cancer Biology, National Cancer Institute, Cairo University, Giza, Egypt; 3 Botany and Microbiology Department, Faculty of Science, Helwan University, Cairo, Egypt

**Keywords:** *Aspergillus niger*, Antimicrobial, Cytotoxicity, Anticancer, GC-mass spectroscopy, Soil fungi

## Abstract

Ten fungal species were isolated from soil in the Western Desert and Wadi El-Natron in Egypt. All fungal isolates were morphologically recognized down to the species level. Methanol extracts of fungal mycelia and ethyl acetate extracts of culture filtrate from the isolated fungi were evaluated for antimicrobial activity against six pathogenic bacteria and one pathogenic yeast (*Candida albicans* ATCC20231). Only ethyl acetate extracts of *Fusarium circinatum*, *Aspergillus niger*, and *Aspergillus terreus* culture filtrates showed significant antimicrobial activity against the majority of the investigated pathogens. The culture filtrate extract of *Aspergillus niger* exhibited notable cytotoxicity towards the breast cancer (MCF-7) cell line, with the lowest detected IC_50_ recorded at 8 μg/μl. Whereas *Fusarium circinatum* and *Aspergillus terreus* had IC_50_s of 15.91 μg/μl and 18 μg/μl, respectively. A gas chromatography-mass spectroscopy (GC–MS) investigation of *A. niger’*s potent extract revealed 23 compounds with different biological activities. Glycidyleoleate was found to be the main extract component. *Aspergillus niger* extract was chosen to study its possible cytotoxic mechanism. The extract was found to induce apoptosis and cell cycle arrest at the < 2n stage. Despite a significant increase in caspases 8 and 9, the production levels of tumor necrosis factor α (TNF-α) and interleukin 6 (IL-6) have shown a significant decrease. The high interaction of glycidyleoleate against the studied cytokines’ binding receptors was demonstrated via docking studies. In conclusion, the available data revealed that the culture filtrate extract of *A. niger* possesses promising antimicrobial, cytotoxic, and immunomodulatory properties.

## Introduction

Many microbial species have the ability to adapt to alterations in their environment through the production of natural molecules as a means of survival in harsh environments, and soil is a rich supply of these microbes. A potential source of therapeutic medicines is the unique metabolites generated by microbes in harsh soil, such as those found in deserts with harsh environmental conditions (Conrado et al. 2022; Bhat et al. 2023).

The creation and development of new pharmaceuticals heavily rely on natural products. They appear to be a reliable supply of biologically significant molecules that serve as a valuable commercial resource for a variety of industries (Bhat et al. 2023). The majority of drugs used today for clinical, pharmacological, and biological objectives are bioactive chemicals obtained from natural sources. More than 60% of the anticancer medications are derived from natural products, demonstrating the abundance of sources from which different medications might be synthesized (Mohamed et al. 2022a, b).

Recently, millions of individuals have died without receiving adequate treatment as a result of severe microbial diseases brought on by harmful, resistant pathogens. The rise in drug-resistant infectious microbial diseases is caused by the overuse of synthetic pharmaceuticals that pollute soil and water (Niazi et al. 2023).

Around 10 million people died from cancer globally in 2020, making it one of the leading causes of mortality (World Health Organization 2022), according to a World Health Organization report released in February 2022 (https://www.who.int/news-room/fact-sheets/detail/cancer (accessed on June 10, 2023)). Today, surgery, radiation therapy, immunotherapy, hormone therapy, targeted therapy, photodynamic therapy, and chemotherapy are the most frequently used cancer treatments (https://www.cancer.gov/about-cancer/ treatment/types (Accessed June 16, 2023). The latter is the most effective treatment choice, but new treatments provide that cancer cells have acquired resistance to it (Wang et al. 2019).Therefore, it is still a desirable field to find new anti-cancer chemicals that can be employed as chemotherapeutic medicines (Li et al. 2021). Therefore, the intrinsic antioxidant activity of fungal secondary metabolites may considerably aid in the development of cancer therapeutic techniques (Sahu et al. 2020).

42% of all natural compounds generated by microorganisms are thought to be produced by fungi. In light of the possibility that their metabolites might replace health security, they are thus seen as one of the key elements of microbial manufacturing companies (Mohammed et al. 2021). Due to their remarkable capacity to take on a variety of shapes in response to unfavorable conditions, fungi thrive in soil and make up 500 to 5000 kg of all soil creatures' biomass per hectare (Sylvia 2005).

Fungi have been regarded as a prospective supply for the discovery and development of unique biologically active metabolites due to their ability to synthesize a diverse array of secondary metabolites (Hashem et al. 2023). Fungi, which make up the majority of eukaryotes, are capable of producing substances with a wide range of structural characteristics that fall under the categories of azaphilones, cytochalasans, macrolides, anthracenones, and naphthalenones. Among the substances discovered in fungi are cytochalasin E, hypothemycin, demethoxyviridin, fussicocin A, destruxin B, fumagillin, and radicicol. Although the focus has largely been on the antibacterial properties of fungal metabolites, fungi have a significant capacity to create toxic secondary metabolites in response to predators, UV radiation, and competition from other bacteria. The polyketide synthase pathway in fungi is known to yield many scaffolds with anticancer action (Shevkar et al. 2022).

About 378 *Aspergillus* species have been identified, of which approximately 180 are important for pharmacological and commercial purposes, according to the World Data Centre for Microorganisms (WDCM) (El-hawary et al. 2020; Gill et al. 2023). Because of its diversity, *Aspergillus* is still one of the most important producers of intriguing secondary metabolites with anti-inflammatory, anticancer, antibacterial, and antioxidant properties (Mokhtar et al. 2023). *Aspergillus oryzae* may have anti-oncogenic properties on the human breast cancer MCF-7 cell line (Plnsn and Siddalingeshwara 2014). It is generally known that members of *Aspergillus* produce a variety of secondary metabolites with different chemical compositions, such as pyranones, alkaloids, cyclopenta peptides, polyketides, sterols, etc., which act as antibacterial, anticancer, antioxidant, antiviral, and other functions (Wei et al. 2022).

The current work aimed to isolate soil fungi from unique Egyptian soil habitats in order to get fungal isolates that may produce novel natural bioactive metabolites with antimicrobial, anticancer, and immunomodulatory activity.

## Materials and methods

### The studied soil samples

Different samples of soil were obtained from the Western Desert (Latitude: 25° 30′ 59.99" N, Longitude: 29° 09′ 60.00" E) (rhizosphere soil surrounding clover plants) and Wadi El-Natrun (Latitude: 30° 24′ 59.99" N, Longitude: 30° 19′ 60.00" E), Al-Beheira Governorate, Egypt, in October 2017. Soil samples were stored in the mycological laboratory of the Helwan University Faculty of Science Botany and Microbiology Department, Egypt. At a depth of 10–15 cm below the earth’s surface, soil samples were collected from each location and transported in sterile, clean polythene bags to the lab. The plant remains were eliminated, and soil samples were dried, crushed, and sieved to remove heavy soil particles before fungi were isolated.

### Isolation and morphological identification of soil fungi

A weight of 1.0 g of soil sample obtained from the different locations was mixed with 100 ml of sterilized distilled water with shaking, and then sixfold serial dilutions were followed (Parkinson and Williams 1961). One ml of each dilution was spread onto plates containing potato dextrose yeast extract agar (PDYA) media, including (g/L): potato 300, dextrose or glucose 20, yeast extract 1.5, agar 15, distilled water 1L, supplemented with ampicillin (50 ug/mL) to prevent the growth of bacteria (Atlas 2010).

The plates were kept at 25°C. The fungal colonies were purified and cultured on PDA slants at 25°C for 5 days. After that, they were stored at 4°C in a refrigerator. Using the identification keys supplied by the following literature (Barnett and Hunter 1972), isolates were morphologically identified to the genus level according to their morphological traits and microscopic examination at the Mycological Center (AUMC), Assiut University, Egypt.

### Cultivation of isolates for production of secondary metabolites

Before being sliced into 5 mm plugs, the fungal strains were cultured on PDA medium for 7 days. Two plugs of active mycelia were inoculated into a 50-ml PDY broth medium made out of 1 L of potato infusion, 20 g of dextrose, and 2 g of yeast extract in 250-ml Erlenmeyer flasks (Janli et al. 2017).

The flasks were then incubated for 7 days at 25°C under static conditions. Filtration was carried out to separate fungal biomass from culture filtrate. After that, using distilled water, the mycelia were properly washed and then dried. The obtained fungal mats and culture filtrate were stored in sterile conditions at 4°C for further studies.

### Secondary metabolites extraction

#### Metabolite extraction from fungal mycelia

The intracellular metabolites were prepared using dried and homogenized mycelia (5 g) of each isolated fungus. The mycelial extracts were extracted by employing an equal ratio of 2:2:1 methanol: chloroform: distilled water (Hamad et al. 2022). The hydrophilic upper layer was collected and dried under a vacuum for 24 h before use.

#### Metabolite extraction from culture filtrate

To extract extracellular metabolites from the culture filtrate, a 1:1 volume-to-volume ratio of ethyl acetate was mixed with the culture filtrate and vigorously shaken by hand for a duration of 1 h. The organic phase was removed and subjected to evaporation until complete dryness using a rotary evaporator (IKA rotary evaporator, Staufen, Germany) (Ahmad et al. 2017).

### Preliminary bioactivity screening

#### Antimicrobial activity

A total of six pathogenic bacteria, comprising both Gram-positive and Gram-negative types, including *Staphylococcus aureus ATCC25923, Micrococcus luteus* ATCC 9341*, Streptococcus pneumonia* ATCC49619*, Escherichia coli* ATCC25922*, Pseudomonas aeruginosa* ATCC7853*, and Proteus mirabilis* ATCC29906, were subjected to the crude extracts for antibacterial activity evaluation. In this study, *Candida albicans* ATCC 20231, a yeast isolate, was employed to evaluate its antifungal efficacy. The well-diffusion approach was employed to evaluate the antibacterial properties of crude extracts (El-Sayed et al. 2023).

Briefly, the tested microbial pathogens were pre-cultured in nutrient broth medium for 24 h at 37°C. Then, 100 μl of 24-h cultures (1 × 10^6^ CFU/mL) were plated onto petri plates with 20 ml of nutrient agar medium. Each agar plate was divided into 6 wells (5 mm in diameter), and 100 μl of fungal crude extracts (10 μg/ml) were added.

Subsequently, the plates were subjected to incubation at a temperature of 4°C overnight to allow for the diffusion of the extracts. This was followed by a subsequent incubation at a temperature of 37°C for 24–48 h. In order to evaluate the antibacterial efficacy, measurements of the diameter of the inhibitory zone surrounding the well were conducted. The positive controls in this study consisted of antibacterial and antifungal drugs, specifically gentamicin at a concentration of 10 μg/disc and amphotericin B at a concentration of 100 units/disc. On the other hand, the negative controls were composed of 100 μl of a mixture of water and methanol in a 2:1 volume-to-volume ratio, or ethyl acetate (Mokhtar et al. 2023). The antimicrobial assay was carried out in triplicate.

### Cytotoxicity evaluation

#### Cell line and culture conditions

The cytotoxicity of the fungal extracts, which exhibited antimicrobial activity, was evaluated against the MCF-7 human breast cancer cell line with the MTT technique. The breast cancer cell line was cultivated and preserved in the Biochemistry Unit, Cancer Biology Department, National Cancer Institute, Cairo University, Egypt. The cells were cultured as a monolayer in a 25-cm^2^ flask containing 7 ml of Roswell Park Memorial Institute (RPMI-1640) medium supplemented with 10% foetal bovine serum (FBS). The cells were then incubated under standard laboratory conditions (Hamad et al. 2022).

#### In vitro* cytotoxic assay*

A volume of 100 μl of MCF-7 cells at a concentration of 2 × 10^4^ cells/ml was added to individual wells of a sterile 96-well flat-bottom plate. The cells were incubated for 24 h, after which they were exposed to varying concentrations of culture filtrate extracts from different fungi (ranging from 5 to 50 mg/mL). Each sample was treated in triplicate. Furthermore, a volume of 100 μl of the MCF-7 cell line was maintained in triplicate as an untreated negative control. The plates were subjected to incubation for 24 h at a temperature of 37°C in a 5% carbon dioxide incubator. The cells were thereafter exposed to 10 μl of MTT solution (5 mg/1ml of 1.0 M PBS, pH 7.4) for a duration of 4 h at 37°C. The medium was removed, and subsequently, 100 μl of dimethyl sulphoxide was added to each well. The solution was carefully mixed using a pipette and subsequently cultured for a duration of 2 h at a temperature of 37°C within a 5% CO_2_ incubator (Hamad et al. 2022).

The ELISA plate reader (Model ELX800, BioTek Instruments, Inc., Winooski, VT, USA) was employed to measure the absorbance of each well at a wavelength of 570 nm. The IC_50_ values for each extract were determined using GraphPad Prism 7 software (GraphPad Software, La Jolla, CA, USA). The percentage of cell viability was assessed by employing the following equation, in which the treated cells were compared to the control cells:

$$\mathrm{Cell viability }(\mathrm{\%})=(\mathrm{Absorbance of treated cells})/ (\mathrm{Absorbance of control cells}) * 100$$

According to the primary screening, the *A. niger* culture filtrate extract was the most effective and had the lowest IC_50_, so it was chosen for further studies.

### Molecular characterization of the most potent isolate

#### DNA isolation and polymerase chain reaction (PCR) technique

A week-old PDA culture's fungal mycelium was used to extract the whole genomic DNA using the Quick-DNA™ Fungal/Bacterial Miniprep Kit (ZYMO RESEARCH) (Prabha et al. 2013). The extracted DNA was analyzed using a 1% agarose gel obtained from Sigma-Aldrich. The gel was prepared using 1X TAE buffer, which consists of tris–acetate-ethylenediaminetetraacetic acid, and it contained 0.5 mg/ml of ethidium bromide (Sigma-Aldrich). The visualization of the ethidium bromide-stained gel was achieved by employing a UV transilluminator manufactured by Vilber Lourmat, a company based in Collégien, France. The PCR reactions were conducted using COSMO PCR RED Master Mix (W1020300X) from Birmingham, England, in accordance with the manufacturer’s instructions. The ribosomal internal transcribed spacer was amplified using the internal transcribed spacer 1 (ITS1) and ITS4 primers, and all reactions were carried out in a reaction volume of 50 μL. The amplification reactions were conducted in a Thermal Cycler (Biometra, Germany) according to the following procedure: The first cycle consisted of a 3-min period at 95°C for initial denaturation. This was followed by 35 cycles, each consisting of 20 s at 95°C for denaturation, 20 s at 55°C for annealing, and 30 s at 72°C for extension. After the cycles, a final extension step was performed for 10 min at 72°C. The reaction was then kept at 4°C. Using an ITS4 primer from Eurofins Genomics (previously GATC Biotech; Ebersberg, Germany) and an ABI 3730xl DNA sequencer, the purified material utilizing the Zymo-Spin™ method was sequenced using the Sanger method (El-Sayed et al. 2022).

#### Sequencing analysis

The blast was conducted using Geneious Pro software 11.1.5 for the obtained nucleotide sequence. In contrast, MUSCLE techniques were used to align the highly similar sequence that was retrieved from the National Center for Biotechnology Information (NCBI) (https://blast.ncbi.nlm.nih.gov/Blast.cgi, accessed on October 25, 2023). Maximum likelihood techniques were used to build the phylogenetic tree (El-Sayed et al. 2022).

### GC–MS analysis of A. niger extracellular secondary metabolites:

The most potent fungal extract, found in the culture filtrate of *A. niger* extract, was employed in the current work to pinpoint the bioactive secondary metabolites. The following features apply to the gas chromatography–mass spectrometry system utilized for the GC–MS analysis: a TRACE GC Ultra Gas Chromatography with an ISQ Single Quadrupole Mass Spectrometer as the detector (THERMO Scientific Corp., USA). The GC–MS system came with a TR-5 MS column (30 m × 0.32 mm i.d., 0.25 m film thickness). For the investigations, helium was used as the carrier gas. It flowed at a rate of 1.0 mL/min and had a split ratio of 1:10: 60°C for 1 min; ascend at 4.0°C/min to 240°C and hold for 1 min. The detector and injector were both kept at 210°C. The mixes (diluted 1:10 hexane/v/v) were always injected into a volume of 1L. In order to study mass spectra with a spectral range of m/z 40–450, electron ionization (EI) at 70 eV was utilized. The chemical components of the metabolites were determined by their retention indices (relative to n-alkanes C8-C22) and mass spectrum matching to authentic standards using AMDIS software (www.amdis.net) (when available), the Wiley spectral library collection, and the NSIT library database.

### Detection of cell cycle using cytell™ cell imaging system

The Cytell™ Cell Cycle Kit (GE Healthcare Japan, Tokyo, Japan) was used to dye MCF-7 cells for 24 h at 37°C and 5% CO_2_. The cells were subsequently evaluated using a Cytell^TM^ cell imaging system (GE Healthcare Japan) (Hamad et al. 2022).

### Apoptosis analysis

*A. niger* culture filtrate extract IC50 (8µg/mL) was applied to MCF-7 cells (1 × 10^6^/well) 24 h before the experiment. Cells that were not treated served as a negative control. According to the manufacturer’s recommendations, the TACS® Annexin V-FITC Apoptosis Detection Kit was used to track apoptosis and living cells. The ratio of live to apoptotic cells was measured using the Cytell™ cell imaging technique (GE Healthcare Japan) (Hamad et al. 2022).

### Assessment of caspases levels

The effect of *A. niger* culture filtrate extract on the levels of caspases 8 and 9 in the MCF-7 cell line culture was investigated at its IC_50_ concentration using the Caspase 8 Human ELISA kit (BMS2024) and Caspase 9 Human ELISA kit (BMS2025) using a spectrophotometer (Tecan Group Ltd., Seestrasse, Männedorf, Switzerland). The levels of caspases 8 and 9 were measured at 450 nm against doxorubicin (positive control) and untreated cells (negative control) according to standard protocols of the manufacturer.

### ELISA estimation of interleukin-6 (IL-6) and TNF-α cytokines

IL-6 and TNF-α were measured in MCF-7 cells treated with *A. niger* culture filtrate extract at an 8 μg/mL concentration and incubated for 24 h at 37°C in a 5% CO_2_ incubator using an ELISA kit (Sunlong Biotech, China) as directed by the manufacturer (Hamad et al. 2022).

#### In silico docking study

In this study, the impact of glycidyloleate (C_21_H_38_O_3_), the major compound in the *A. niger* culture filtrate extract, on the pro-inflammatory cytokines IL-6 and TNF-α, was investigated using computational methods. The structures of the two cytokines, human IL-6 (ID: 1alu) (Somers et al. 1997) and human TNF-α (ID: 2az5) (He et al. 2005), were acquired from the Protein Data Bank (PDB), respectively. The molecular docking technique was performed utilizing the Molecular Operating Environment application (MOE 2014.09). The energy of the ligand, namely the glycidyloleate molecule, was minimized. Subsequently, the appropriate sequence of both cytokines was selected, followed by protonation. Finally, the partial charges were estimated.

### Statistical analysis

The analysis of the data was carried out according to (Snedecor and Cochran 1991). Statistical analysis was performed using one-way analysis of variance (ANOVA), followed by Duncan’s multiple comparison test using IBM Statistical Version 21 at *P* < 0.05, which was denoted as being statistically significant for the compared means using the least significant difference (LSD at the 5% level).

## Results

### Fungal isolates

Ten fungal isolates were found in soil samples collected from certain geographic areas in Egypt. The soil samples were gathered from the Western Desert, El-Dakhla Oasis (the soil rhizosphere around the clover plant), and Wadi El-Natrun. Based on their morphological and microscopic properties, all isolates were identified at the species level at the Assiut-University Mycological Center (AUMC), Egypt. *Penicillium*,* Aspergillus*, and *Fusarium* genera were the dominant isolates. The isolated and identified fungal species were summarized in Table 1.

**Table 1:** Fungal species isolated from the studied Egyptian habitats

Fungal code	Region	Fungal isolate
1	Western Desert, El-Dakhla Oasis (Region 1)	*Fusarium circinatum* Nirenberg& O’Donnell
2		*Fusarium subglutinans* (Wollenweber &Reinking) Nelson et al
3		*Aspergillus niger*van Tieghem
4		*Aspergillus quadrilineatus* Thom &Raper
5		*Cochliobolus spicifer* Nelson
6		*Penicillium chrysogenum* Thom
7		*Penicillium crustosum* Thom
8		*Fusarium pseudocircinatum* Nirenberg& O’Donnell
9		*Penicillium aurantiogriseum* Dierckx
10	Wadi El-Natrun ( Region 2)	*Penicillium crustosum* Thom
11		*Aspergillus terreus* Thom

*Penicillium* and *Fusarium* were the dominant genera and made up 33% of the isolated colonies in soil samples from the Western Desert. *Aspergillus* made up 22% of isolated colonies, while *Cochliobolus* made up 11% of all isolated colonies. *Penicillium* and *Aspergilli* predominated by 50% in the soil samples from Wadi El-Natrun.

### Screening of the antimicrobial activity of the fungal isolates

Different extracts from the mycelia and culture filtrate of the investigated fungi were evaluated for their antimicrobial potency against a range of reference pathogenic bacteria and yeast. The polar mycelial extract of the tested fungi had no antimicrobial activity against all tested pathogens. Only ethyl acetate extracts of *F. circinatum, A. niger,* and *A. terreus* culture filtrates showed significant antimicrobial activity against the majority of the investigated pathogens.*F. circinatum* culture filtrate extract demonstrated significant antimicrobial activity against *S. pneumonia* (34.0 ± 0.09 mm*), P. aeruginosa* (28.3 ± 0.22 mm), *E. coli* (28.0 ± 0.13 mm), *C. albicans *(17.0 ± 0.12 mm), and *S*. *aureus* (16.7 ± 0.17mm) as compared to the studied standard antibiotics*. A. niger* culture filtrate extract showed antimicrobial activity against *E. coli* (23.5 ± 0.06 mm), *S. pneumonia* (18 ± 0.11 mm*), P. aeruginosa* (17.0 ± 0.18mm), and *S*. *aureus* (10.5 ± 0.08mm), while *A. terreus* culture filtrate extract revealed antimicrobial activity against *P. aeruginosa* (21.5 ± 0.04), *S. pneumonia* (20 ± 0.15 mm*),* and *S*. *aureus* (14.7 ± 0.19mm)*. P. mirabilis* and *M. luteus* were resistant to all culture filtrate extracts (Table 2).

**Table 2:** Antimicrobial activity of the culture filtrate extracts of the isolated fungi

Test organism Isolates	Inhibition zone size (mm)						
	Gram- positive bacteria			Gram- negative bacteria			Pathogenic yeast
	*S.* *aureus*	*M. luteus*	*S.* *pneumoniae*	*P. mirabilis*	*E.* *coli*	*P.* *aeruginosa*	*C. albicans*
*Fusarium circinatum*	16.7 ± 0.17	‒	34.0 ± 0.09	‒	28.0 ± 0.13	28.3 ± 0.22	17.0 ± 0.12
*Fusarium subglutinans*	‒	‒	‒	‒	‒	‒	‒
*Aspergillus niger*	10.5 ± 0.08	‒	18 ± 0.11	‒	23.5 ± 0.06	17.0 ± 0.18	‒
*Aspergillus quadrilineatus*	‒	‒	‒	‒	‒	‒	‒
*Cochliobolus spicifer*	‒	‒	‒	‒	‒	‒	‒
*Penicillium chrysogenum*	‒	‒	‒	‒	‒	‒	‒
*Penicillium crustosum*	‒	‒	‒	‒	‒	‒	‒
*Aspergillus terreus*	14.7 ± 0.19	‒	20 ± 0.15	‒	‒	21.5 ± 0.04	‒
*Penicillium* *crustosum*	‒	‒	‒	‒	‒	‒	‒
*Fusarium pseudocircinatum*	‒	‒	‒	‒	‒	‒	‒
*Penicillium* *aurantiogriseum*	‒	‒	‒	‒	‒	‒	‒
Gentamicin	21.4 ± 0.08	18.4 ± 0.22	27.2 ± 0.60	20.0 ± 0.10	19.3 ± 0.15	22.41 ± 0.08	Nt
Amphotericin B	Nt	Nt	Nt	Nt	Nt	Nt	21.2 ± 0.10

Values for the growth inhibition zone (estimated as the clear zone diameter around the well) are means ± SD of three replicas. The well diameter (5 mm) is included “-” indicates no inhibition. Nt: not tested.

### Cytotoxic activity

**Fig. 1:**
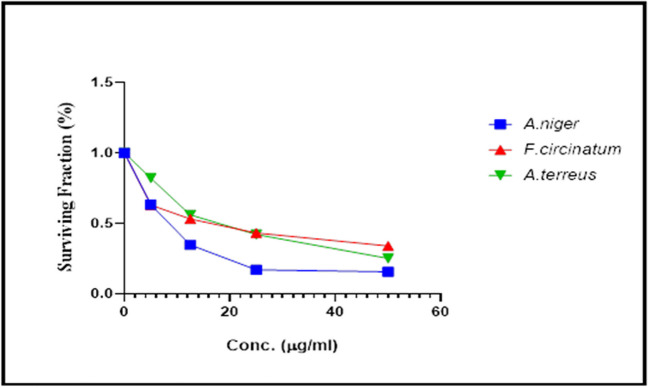
Viability response graph of MCF-7 cells exposed to varying concentrations of culture filtrate extracts derived from *A. niger, F. circinatum*, and *A. terreus*

The cytotoxicity of *F. circinatum, A. niger, and A. terreus* isolates’ culture filtrates’ extracts was tested against a human breast cancer cell line (MCF-7). Cytotoxicity was evaluated and represented as a survival fraction as compared to untreated control cells. A variety of concentrations were used to calculate the IC_50_ and determine cell viability. Viability was shown to be dose-dependent. The treatment of MCF-7 cells with different concentrations of culture filtrate extracts from *A. niger, F. circinatum,* and *A. terreus* dramatically inhibited cell growth with IC_50_ values of 8 μg/μl, 15.91 μg/μl, and 18 μg/μl, respectively (Fig. 1). The findings indicated that *A. niger* culture filtrate extract had the lowest IC_50_ and had the greatest promise for further research into its anticancer mechanisms. So, it was chosen to complete the current investigation.

### Molecular characterization of the most potent fungal isolate

**Fig. 2:**
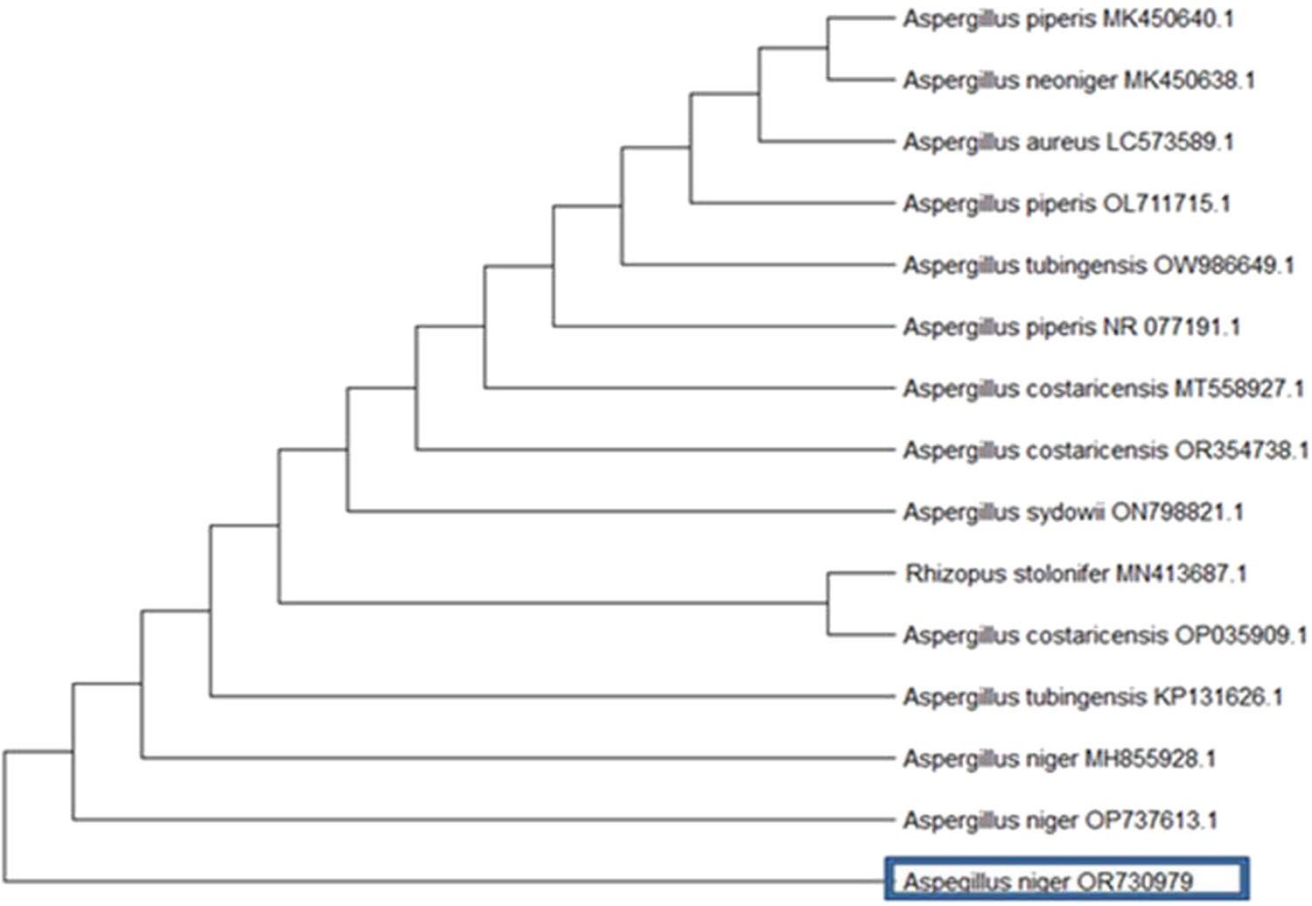
Aspergillus* niger* molecular identification. The phylogenetic tree was created by the Geneious Pro program using the maximum likelihood technique

The nucleotide sequence derived from fungal DNA was subjected to analysis utilizing the GenBank database, employing advanced BLAST (Megablast) searches provided by the National Center for Biotechnology Information (NCBI). The fungal isolate was taxonomically classified as *A. niger* and subsequently deposited in the Genbank database under the designation *A. niger*, accompanied by the unique fungus identification code OR730979. Moreover, the constructed phylogenetic tree, which includes the partial sequence of *A. niger* OR730979 along with related sequences from other fungi available in GenBank, revealed a significant similarity of 88% between *A. niger* OR730979 and OP737613 (Fig. 2).

### Metabolic profile of the culture filtrate extract of the most potent isolate, A. niger, by the GC-mass technique

**Fig. 3:**
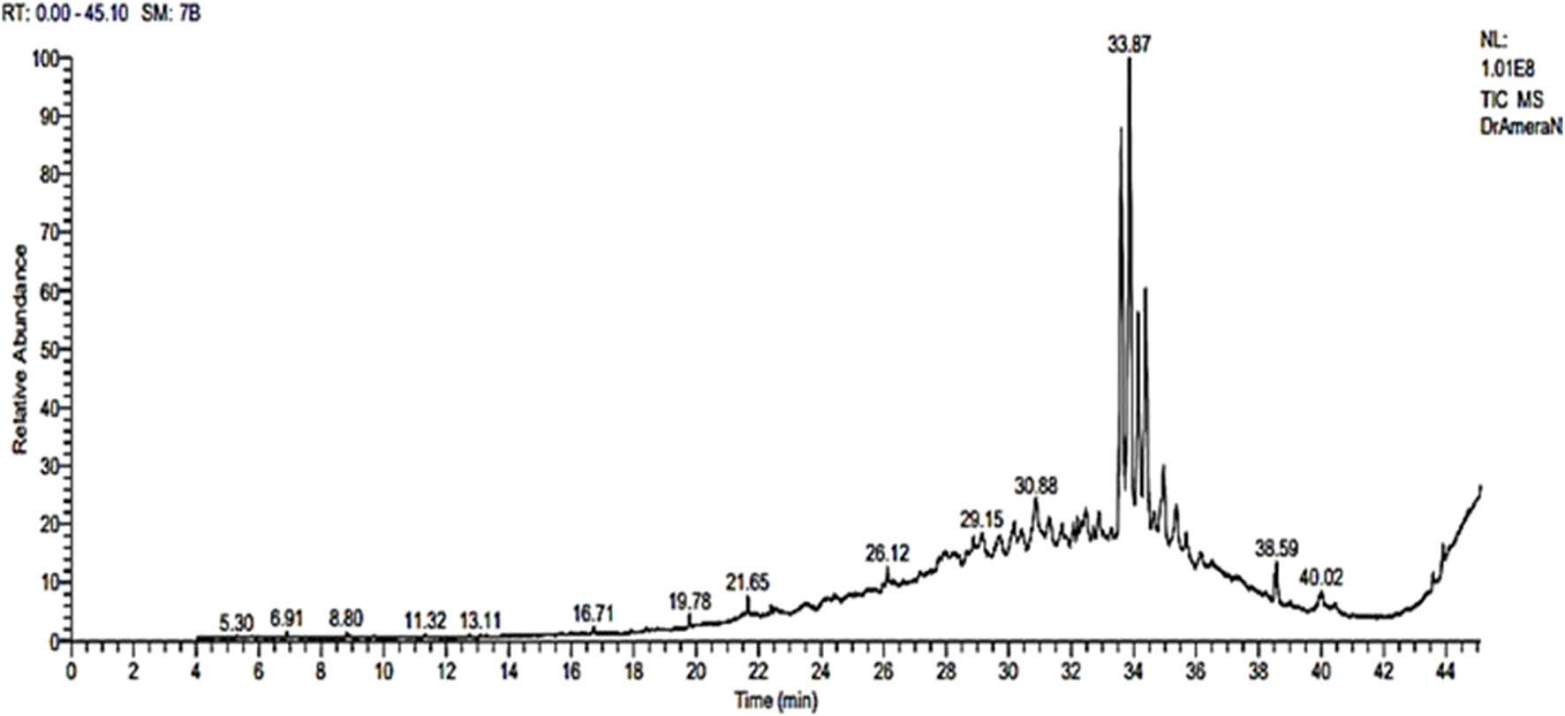
GC–MS chromatogram of *A. niger* culture filtrate ethyl acetate extract

The metabolic composition of the ethyl acetate extract derived from the culture filtrate of *A. niger* was examined through the utilization of GC/MS. The primary metabolites were glycidyloleate, heptacosane, Docosane,11-decyl-, dotriacontane, and 9-Octadecenoic Acid (Z)-, with peak areas of 22.76, 1.77, 1.14, 1.13, and 1.02%, respectively. Twenty-three metabolites were successfully detected. The retention time (RT) of these compounds ranged from 6.91 to 44. The major compound detected was glycidyloleate, with an area percent of 22.76. The chemical formula, molecular weight, retention time, and peak area (%) of the non-polar metabolites were shown in Table 3 and Fig. 3.

**Table 3:** GC/MS-Based metabolites from *A. niger* culture filtrate extract

NO	R.T	M.F	Compound Name	Molecular Formula	Molecular weight	Area%
1	6.91	925	Cyclohexene, 1-methyl-4-(1-methylethenyl)-, (S)-	C10H16	136	0.15
2	8.80	893	Tetradecane	C14H30	198	0.22
3	16.71	861	3-Trifluoroacetoxypentadecane	C17H31F3O2	324	0.23
4	19.78	975	3,4-Dihydro-2H-1,5-(3"-T-Butyl)benzo dioxepine	C13H18O2	206	0.40
5	21.65	924	1-Decanol, 2-hexyl-	C16H34O	242	0.74
6	22.39	802	1,3,5-Triazine-2,4-diamine, 6-chloro-n-ethyl	C5H8CLN5	173	0.22
7	24.10	811	Tetrapentacontane, 1,54-dibromo	C54H108Br2	914	0.69
8	25.97	805	14-á-H-pregna	C21H36	288	0.28
9	26.12	837	1-Heptacosanol	C27H56O	396	0.67
10	27.17	811	Tetrapentacontane, 1,54-dibromo-	C54H108Br2	914	0.42
11	27.78	831	Heptacosane	C27H56	380	0.31
12	27.95	810	Ethanol, 2-(octadecyloxy)-	C20H42O2	314	0.48
13	28.87	834	Butyl dotriacontyl ether	C36H74O	696	0.41
14	29.15	823	Docosane,11-decyl-	C32H66	450	1.14
15	30.10	832	Octatriacontylpentafluoropropionate	C41H77F5O2	696	0.66
16	30.88	840	Heptacosane	C27H56	380	1.77
17	33.87	882	Glycidyloleate	C21H38O3	338	22.76
18	36.15	814	Dotriacontane	C32H66	450	1.13
19	38.51	839	12-Methyl-E,E-2,13-octadecadien-1-ol	C19H36O	280	0.81
20	40.01	826	9-Octadecenoic Acid(Z)-	C18H34O2	282	1.02
21	43.59	762	4H-1-Benzopyran-4-ONE, 2-(3,4-dimethoxyphenyl)-3,5-dihydro XY-7-methoxy	C18H16O7	344	0.54
22	43.89	773	1-Heptatriacotanol	C37H76O	536	1.04
23	44.00	758	9,12-Octadecadienoic acid (Z,Z)-, 2,3-Bis[(Trimethylsilyl)oxy]proxyl ester	C27H54O4Si2	498	0.25

### MCF-7 cell cycle arrest by *A. niger* culture filtrate extract

**Fig. 4`:**
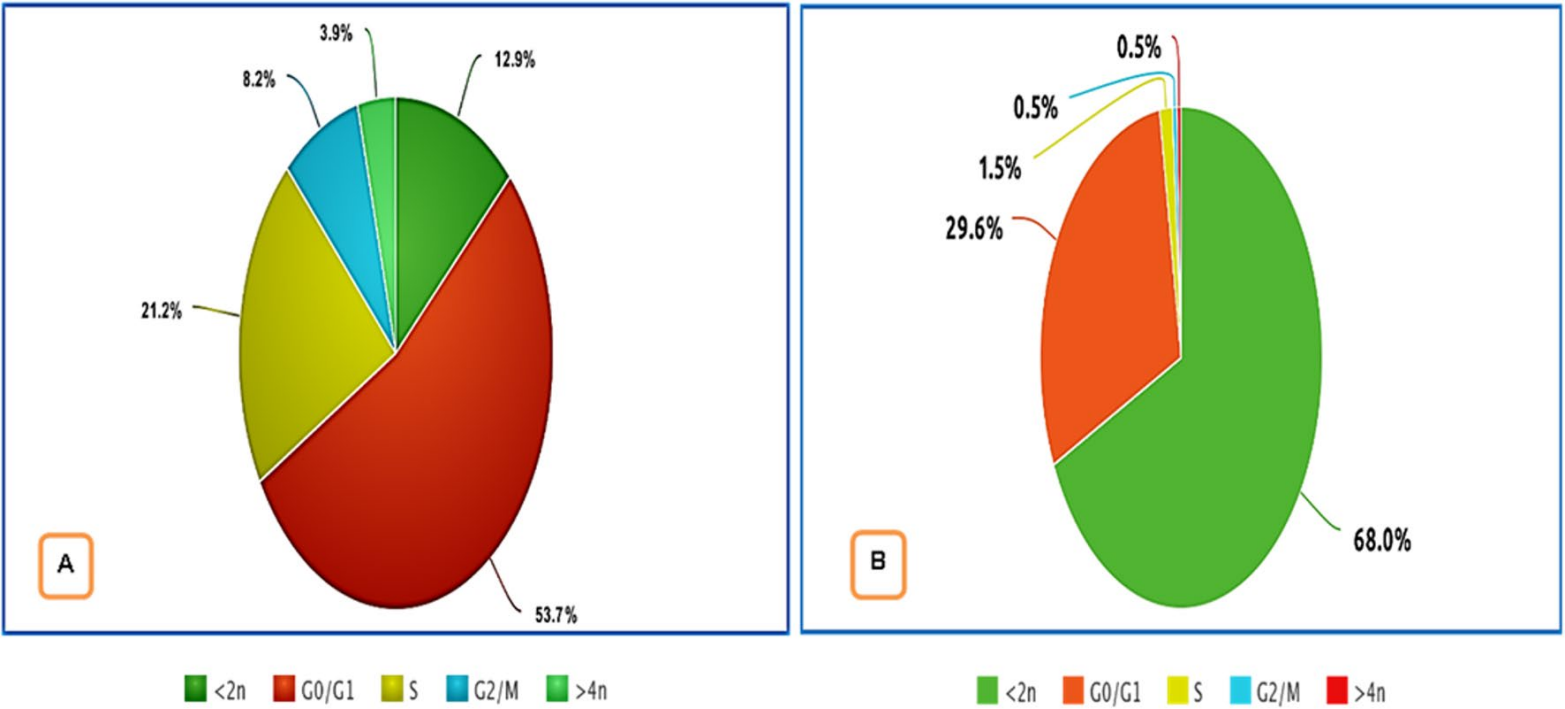
The action of A.* niger* culture filtrate extract (8 µg/mL) on cell cycle phases against the control untreated MCF-7 cell line. Culture filtrate extract of *A. niger* promoted cell cycle arrest in the (< 2n) stage

With the use of the Cytell™ cell imaging apparatus, the pattern of cell cycle stages was analyzed in order to understand how the *A. niger* culture filtrate extract affected the MCF-7 cell cycle. The percentage of cells in the sub-G1 phase (apoptosis-inducing cells) significantly increased after treatment with *A. niger* culture filtrate extract (8 µg/mL), reaching 67.98% compared to 12.94% in the control, while the percentage of cells in the other cell cycle phases decreased to 29.56% for G0/G1 cells, 1.48% for S cells, and 0.49% for G2/M cells, when compared to control untreated cells (G0/G1 phase: 53.73%; S phase: 21.18%; and G2/M phase: 8.24%) (Fig. 4). *A. niger* culture filtrate extract appears to be able to induce sub-G1 phase cell cycle arrest after being cultured for 24 h.

### Apoptosis induction in MCF-7 cells

**Fig. 5:**
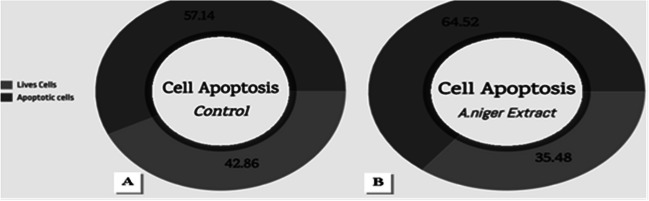
The percentage of apoptotic and live cells of MCF-7, where (A) MCF-7 untreated cell control (B) MCF-7 cells treated with A*. niger* culture filtrate extract (8 µg/mL)

The evaluation of the apoptotic impact on MCF-7 cells, induced by treatment with an extract of *A. niger* culture filtrate at a concentration of 8 µg/mL, was conducted utilizing a Cytell™ cell imaging system and an Apoptosis Detection Kit using Annexin V-FITC. The study's conclusions showed that *A. niger* culture filtrate extract successfully causes apoptosis in MCF-7 cells. The results presented in Fig. 5 indicated a significant rise in the percentage of apoptotic cells, from 57.4 ± 0.1% in the untreated group to 64.52 ± 0.1%.

### Caspases levels evaluation

Compared to control cells (4.88 ± 0.23 ng/mL), the concentration of caspase 8 in MCF-7 cells treated with *A. niger* culture filtrate extract was considerably higher (8.033 ± 0.033 ng/mL). Additionally, the level of caspase 9 in MCF-7 cells treated with *A. niger* culture filtrate extract was significantly increased (24.4 ± 0.56 ng/mL) in comparison to control cells (12.5 ± 0.40 ng/mL) (Table 4). The findings of the study indicated that the extract derived from the culture filtrate of *A. niger* had a significant influence on the levels of caspases 8 and 9 in MCF-7 cells.

**Table 4:** The concentrations of caspase 8 and caspase 9 in untreated cells (control) and MCF-7 treated with *A*. *niger* culture filtrate extract (8 µg/mL)

	**Concentrations (ng/ml)**	
	**Negative control group**	***A. niger* culture filtrate extract treated group (8µg/mL)**
Caspase 8	4.88 ± 0.23	8.033 ± 0.033^*^
Caspase 9	12.5 ± 0.40	24.4 ± 0.56^*^

The data were shown as the average ± standard deviations of three separate replicates. *Statistical significance at *P* < 0.05.

### TNF-α and IL-6 level determination

The findings provided in Table 4 show a significant reduction (*P* < 0.05) in TNF-α production within MCF-7 cells following treatment with *A. niger* culture filtrate extract concentration of 8 µg/mL (138.43 ± 0.43 pg/mL), compared to the control group of MCF-7 cells (269.03 ± 2.98 pg/mL). *A. niger* extract was applied to MCF-7 for 24 h, and this resulted in a substantial decrease in IL-6 production (*P* < 0.05). According to Table 5, untreated MCF-7 cells had an IL-6 concentration of 62 ± 1.3 pg/mL, whereas MCF-7 treated with *A. niger* culture filtrate extract had an IL-6 concentration of 10.5 ± 0.9 pg/mL.

**Table 5:** TNF-α and IL-6 levels of both untreated MCF-7 cells (control) and MCF-7 cells treated with *A. niger* culture filtrate extract (8 µg/mL)

	**Concentrations (pg/mL)**	
	**Negative Control**	***A. niger* culture filtrate extract (8µg/mL)**
TNF-α	269.03 ± 2.98^*^	138.43 ± 0.43
IL-6	62.00 ± 1.3^*^	10.50 ± 0.9

The data were shown as the average ± standard deviations of three separate replicates. *Statistical significance was set at* P* < 0.05.

#### Docking study

**Fig. 6:**
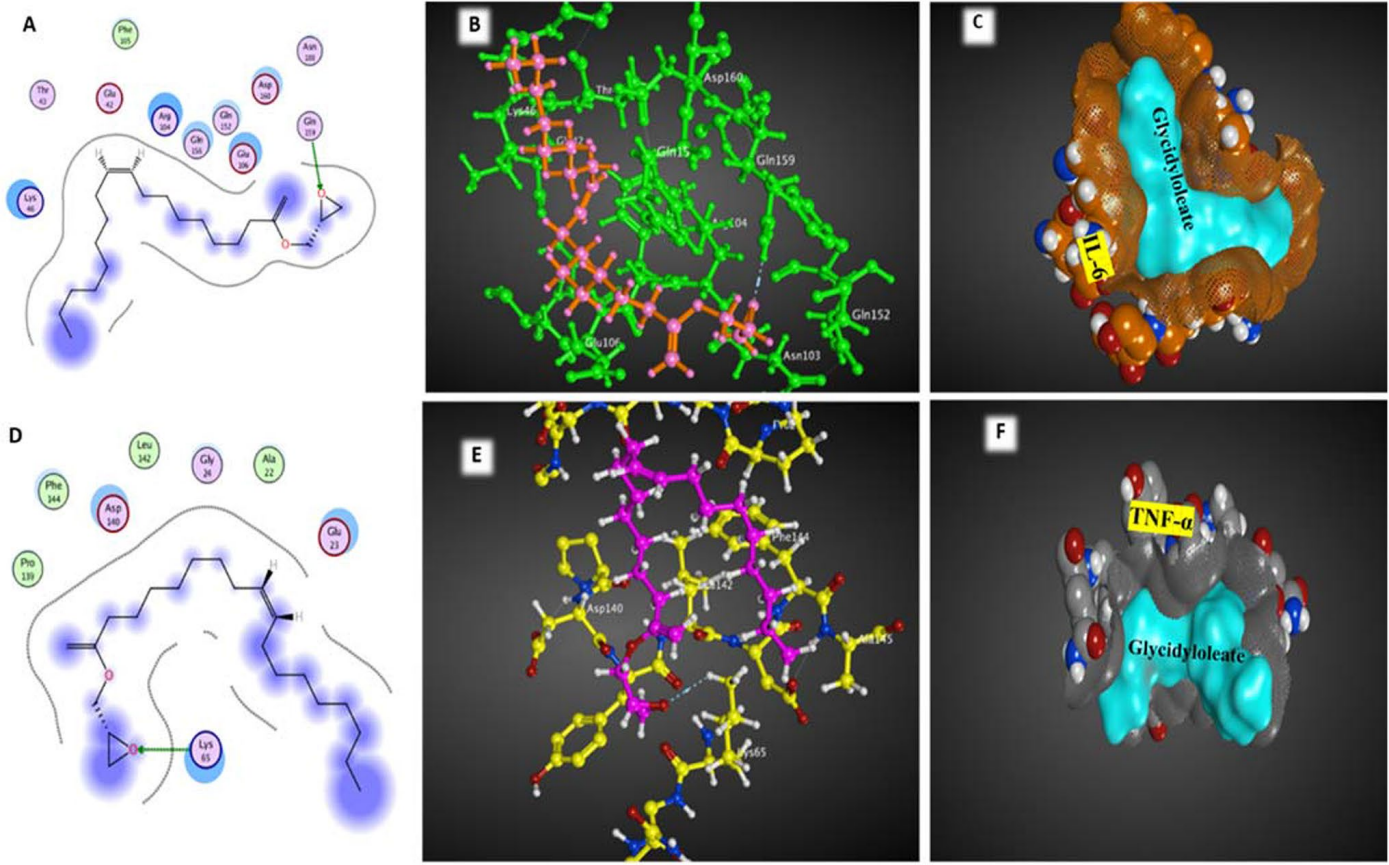
2D and 3D docked interaction map illustrating the binding of the ligand glycidyleoleate to the pro-inflammatory cytokines IL-6 (A, B) and TNF-α (D, E). C and F represent the surface interaction

The cytokines IL-6 and TNF-α, which have pro-inflammatory properties, were chosen as the targets for studying the effects of glycidyloleate, the major metabolite in the potent extract. The efficacy of glycidyloleate in inhibiting the docking interaction with IL-6 and TNF-α receptor binding sites was demonstrated (Fig. 6A, B, C, and D, E, and F, respectively). The application of interaction-free energy was utilized to examine the influence of the ligand on both cytokines. Glycidyloleate exhibited the capability to bind with the above cytokines based on their respective H-interaction scores of -2.0 kcal/mol for IL-6 and -0.7 for TNF-α (as shown in Table 6). The ligand molecule glycidyloleate interacted with the crystal structure of the IL-6 receptor binding site. This showed that glycidyloleate acted as a bridge between the ligand and GLN 159 residues. While the docking results revealed that the ligand compound, glycidyloleate, mediated an interaction with LYS 65 residues of TNF-α.

**Table 6:** In silico docking study of the ligand, glycidyleoleate, with the pro-inflammatory cytokines IL-6 and TNF-α

PDB ID	Docking score (Kcal/mol)	Interaction type	Amino acid residue
IL-6 (1alu)	-2.0	H-acceptor	GLN 159
TNF-α (2az5)	-0.7	H-acceptor	LYS 65

Abbreviations *GLN*: Glutamine amino acid, and *LYS*: Lysine amino acid

## Discussion

The utilization of natural products and their derived components has been extensively utilized in the improvement of human health, with a specific focus on the management of cancer and microbial infections (Niazi et al. 2023). A significant number of these substances are obtained from bacterial and fungal sources.

Yeasts and various fungi, such as *Fusarium*, *Aspergillus*, *Penicillium*, and *Cladosporium*, produce secondary metabolites and enzymes having antimicrobial action (Emmanuel and Igoche 2022). However, the search for new drugs is rising, as antibiotic-resistant pathogens are becoming more common and synthetic drugs with a variety of side effects are becoming more prevalent.

The most important class of organisms on Earth are soil microbes, which provide biologically active metabolites with a wide range of uses that are useful to humanity. Since soil is the source of over 60% of antimicrobial compounds, it is also the most important source for new metabolites with pharmacological and biological action (Niazi et al. 2023). Fungi are primarily potential producers of bioactive substances. According to research, extremophilic fungi have evolved special defenses to withstand extreme conditions such as elevated pressure, temperature, salt, desiccation, and pH levels. These defenses have resulted in the production of novel natural compounds with a variety of biological functions (Zhang et al. 2018). The unique environments of the arid Western desert and the high salinity of Wadi El-Natrun soils were the reasons for choosing these habitats to isolate fungi that may produce natural compounds with different biological properties to survive these harsh conditions. In the current study, soil samples that were taken from the Western Desert and Wadi El-Natrun, two distinctive geographical environments in Egypt, yielded a total of 10 fungal isolates. The three most common isolates came from the genera *Penicillium*, *Aspergillus*, and *Fusarium*.

*A. niger, F. circinatum*, and *A. terreus* culture filtrates were effective against Gram-positive and Gram-negative bacterial strains, according to the findings of the current study's primary screening, indicating their potential as an antibacterial agent. Only *F. circinatum*'s culture filtrate extract had shown antimicrobial activity against *C. albicans*. It has been demonstrated that numerous extracts from soil fungi have antimicrobial properties. For example, a variety of microbes, such as *S. aureus, P. aeruginosa, and C. albicans,* their growth inhibited by the ethyl acetate extract of *A. terreus* (Hamed et al. 2018). Both wild-type and chemically treated isolates of *Aspergillus* species are highly successful at producing secondary metabolites, which effectively suppress the growth of the Gram-positive pathogenic bacteria *B. subtilis* and *S. aureus* (Al-Maqtoofi et al. 2019). According to Xu et al. (2023), *Fusarium* extracts have demonstrated notable antibacterial effectiveness against both Gram-positive bacteria like *S. aureus* and Gram-negative bacteria like *E. coli. Fusarium* extract exhibits potent antimicrobial properties, indicating that it may be utilized as an alternative to antibiotics to treat bacteria that are multidrug resistant.

The cytotoxicity of *F. circinatum, A. niger*, and *A. terreus* was examined against the MCF-7 cell line. The assessment of cytotoxicity was conducted by comparing the survival fraction of cells treated with extract to that of untreated control cells. *A. niger, F. circinatum,* and *A. terreus* culture filtrate extracts significantly reduced breast cancer cell viability, with IC_50_ values of 8 μg/μl, 15.91 μg/μl, and 18 μg/μl, respectively. An earlier investigation demonstrated that *Fusarium* sp. has shown promising cytotoxic effects on various breast and colorectal cancer cell lines (Mohammed et al. 2021). Similar results were observed when HeLa cervical cancer cells were exposed to an ethyl acetate extract of *F. solani* obtained from *Daturametel*, which caused cell death via the mitochondrial pathway (Kuriakose et al. 2014). *A. niger* strain AK-6's ethyl acetate extract demonstrated considerable anticancer properties against the MCF-7 cancer cell line, with an IC_50_ of 102.01 µg/mL (Niazi et al. 2023). *A. niger* CJ6 extract showed moderate potential cytotoxicity in the MIA PaCa-2 cell line (Bhat et al. 2023). According to Sajna et al. (2020), the researchers found that compounds obtained from *A. unguis* AG 1.1 (G) exhibited notable inhibitory effects on the growth and survival of MCF-7, A-431, and COLO-205 cancer cell lines. This inhibition was attributed to the induction of DNA breaks within the cancer cells, leading to the destruction of DNA, stopping the cell cycle, and subsequent cell death (Sajna et al. 2020). According to a different study, *A. aculeatus* secondary metabolites have lethal effects on human breast cancer (MCF-7) and human epidermoid carcinoma (KB) cells (Yodsing et al. 2018). According to Tawfike et al. (2019), the crude extract and active fractions of *A. flocculus* have shown significant anticancer properties against prostate (PC3) and myelogenous leukaemia (K562) cancer cells*.* In the present work, the culture filtrate extract of *A. niger* showed the lowest IC_50_, the most promising bioactivity, and was chosen for further investigations. The potent fungal species has been identified as *A. niger* OR730979 by the application of molecular analysis.

The chemical component of the culture filtrate extract of *A. niger* was analyzed using a GC/MS instrument, resulting in the detection of twenty-three metabolites, most of which exhibit biological efficiency. Glycidyloleate, for instance, has anti-cancer properties (Konovalova et al. 2013). Cyclohexene, 1-methyl-4-(1-methylethenyl)-, (S)- showed anti-inflammatory, antioxidant, antimicrobial, and anticancer activities (Mohamed et al. 2020). Tetradecane was reported to have antifungal and antibacterial activity (Nasr et al. 2022). 1,3,5-triazine-2,4-diamine,6-chloro-N-ethylhad anti-cancer activity (Makowska et al. 2018). Heptacosane had antibacterial activity (Konovalova et al. 2013). 9-Octadecenoic acid (Z) had antioxidant and anticancer properties (Belakhdar et al. 2015).

Cell cycle phase arrest is a frequently recorded incident in response to cytotoxic chemical drugs (Kamat et al. 2020). Utilizing the Cytell™ cell imaging technology, the examination of the pattern of distribution of cell cycle phases was carried out in the current study. The induction of the sub-G1 peak, which demonstrated that the *A. niger* culture filtrate extract treatment caused apoptosis to occur, was the study’s most notable discovery. An earlier investigation found that *A. unguis* mycelial extract exhibited a dose-dependent effect on the G0 peak or sub-G1 peak, representing the proportion of cells undergoing apoptosis (Sajna et al. 2020). Leukemic cells subjected to the fungi-derived Greensporone C showed a similar observation regarding the increase of the apoptotic phase (Prabhu et al. 2018).

Nuclear disintegration, nuclear chromatin condensation, DNA breakage by enzymes, and loss of plasma membrane asymmetry are the hallmarks of the highly coordinated form of cell death known as apoptosis. Molecules that suppress the development of cancer cells by inducing apoptosis might be a realistic mechanistic representation of cancer treatment that avoids undesirable side effects and drug resistance (Majoumouo et al. 2020). The Annexin V-FITC/PI apoptosis detection assay, commonly employed to differentiate between viable cells and those in various stages of apoptosis, was employed to illustrate the induction of apoptosis. The *A. niger* culture filtrate extract was shown to significantly increase the proportion of early and late apoptotic cell populations in MCF-7. This result suggested that apoptotic cell death was induced by the *A. niger* culture filtrate extract. Apoptotic cell death is known to be mediated by the caspase family of cysteinyl proteases.

Caspases are important apoptosis mediators. Extrinsic apoptosis is mediated by caspase-8, and intrinsic apoptosis is started by caspase-9 (Aral et al. 2019). In the current investigation, MCF-7 cells were subjected to an extract of the *A. niger* culture filtrate in order to examine the role of caspase-8 and caspase-9 in the apoptosis that fungal metabolites elicit. The results demonstrated that the *A. niger* culture filtrate extract significantly increased the levels of caspases 8 and 9 in MCF-7 cells, indicating that the extract may be involved in the induction of caspases as apoptosis mediators.

There is growing proof that pro-inflammatory cytokines contribute to the development of cancer. More aggressive cancer cells grew more quickly when the cytokines TNF-α and IL-6 were present (Chung et al. 2017). They had been found in several cell lines and were implicated in cell division, proliferation, and death (Abdellatif et al. 2022). *A. niger* extract at an 8 g/mL concentration was applied to MCF-7 cells for 24 h, during which time IL-6 and TNF-α production were shown to be significantly reduced. A positive result of treatment with the culture filtrate extract was a decrease in these proinflammatory cytokines, which may have led to the MCF-7 cell line's reduced cell division and proliferation. The potential of recognizing and predicting the efficacy of the ligand glycidyleoleate, the major compound in the studied extract, against specific proteins via receptor-ligand interactions can be achieved using molecular docking techniques. The production of cytokines is of utmost importance in the reaction of macrophages to inflammatory stimuli (Elenkov and Chrousos 2002). Macrophages serve as a significant reservoir of various cytokines and growth factors that are stimulated by exogenous molecules. It is significant that an unregulated inflammatory response can lead to the development of persistent and severe chronic inflammation (Lowenstein et al. 1996). Macrophages possess the ability to secrete various inflammatory molecules, such as TNF-α and IL-6, that are pivotal in governing the inflammatory reaction (Salman et al. 2022). The present investigation unveiled a noteworthy interaction between the ligand, glycidyleoleate, and the proteins IL-6 and TNF-α, potentially resulting in a reduction in the concentrations of these cytokines.

## Conclusions

The biological functions of the culture filtrate extract from *A. niger* OR730979, isolated from the Western Desert, Egypt, were evaluated in the current study. The in vitro examination of the ethyl acetate extract derived from the culture filtrate of *A. niger* led us to suggest its potential antimicrobial properties against human pathogenic bacteria. Based on the findings of the GC–MS analysis, 23 bioactive metabolites, which are known to have a variety of therapeutic uses, were found in the sample. In addition, it was shown that the culture filtrate extract derived from *A. niger* exhibited a cytotoxic impact on the MCF-7 breast cancer cell line in a dose-dependent manner. This effect was characterized by the induction of cell death and the generation of both early and late apoptosis. Furthermore, the study demonstrated an elevation in the levels of caspase 8 and 9, while the production levels of TNF-α and IL-6 exhibited a significant reduction. The findings of the study provide avenues for future research that aims to investigate in vivo effects and identify the mechanisms of action. The isolated *A. nige*r showed substantial potential for use in the development of novel antibiotics and anticancer therapies.

## Data Availability

All data used have been included in the manuscript.
